# Carboxylated Osteocalcin as an Independent Predictor of Mean Arterial Pressure and the Atherogenic Index in Adults

**DOI:** 10.3390/ijms26041733

**Published:** 2025-02-18

**Authors:** José Rafael Villafán-Bernal, Jorge David Rivas-Carrillo, Iris Paola Guzmán-Guzmán, Jose Luis Frias-Cabrera, Edgar Alfonso Rivera-León, Raigam Jafet Martinez-Portilla, Sergio Sánchez-Enríquez

**Affiliations:** 1 Molecular Biology and Genomics Department, Centro Universitario de Ciencias de la Salud, Universidad de Guadalajara, Guadalajara 44340, Mexico; jvillafan@inmegen.edu.mx (J.R.V.-B.); edgar.rleon@academicos.udg.mx (E.A.R.-L.); 2 Investigador por Mexico, Secretaria de Ciencias, Humanidades, Tecnología e Innovación (SECIHTI), Mexico City 03940, Mexico; 3 Immunogenomics and Metabolic Diseases Laboratory, Instituto Nacional de Medicina Genómica, SS, Mexico City 14610, Mexico; joseluisfrica@gmail.com; 4 Iberoamerican Research Network in Translational, Molecular and Maternal-Fetal Medicine, Mexico City 01219, Mexico; 5 Physiology Department, Centro Universitario de Ciencias de la Salud, Universidad de Guadalajara, Cuerpo Académico UDG-CA-533, Guadalajara 44340, Mexico; jorge.rivas@academicos.udg.mx; 6 Laboratory of Multidisciplinary Research and Biomedical Innovation, Universidad Autónoma de Guerrero, Chilpancingo 39086, Mexico; pao_nkiller@yahoo.com.mx; 7 Division for Biomedical Sciences, Centro Universitario de los Altos, Universidad de Guadalajara, Cuerpo Académico UDG-CA-1173, Tepatitlán de Morelos 47620, Mexico; 8 Evidence-Based Healthcare Department, Nacional Institute of Perinatology “Isidro Espinosa de los Reyes”, Ciudad de México 11000, Mexico

**Keywords:** osteocalcin, arterial pressure, vascular disease

## Abstract

Bone-derived proteins, including carboxylated osteocalcin (cOC), are thought to play a role in cardiovascular and metabolic health. cOC is recognized for its strong affinity for calcium hydroxyapatite and its possible involvement in vascular calcification and lipid metabolism. Although the undercarboxylated form of osteocalcin (ucOC) has been widely researched, the connections between cOC and cardiovascular risk markers, such as mean arterial pressure (MAP), pulse pressure (PP), and the atherogenic index, are still not well understood. This cross-sectional study comprised 81 adults from Western Mexico; selection was based on rigorous inclusion criteria. Participants underwent various measurements, including anthropometric, biochemical, and cardiovascular assessments, such as the body mass index (BMI), body fat percentage, serum glucose, insulin resistance (HOMA-IR), glycated hemoglobin (HbA1c), lipid profile, creatinine, blood pressure parameters, and the atherogenic index. Serum cOC levels were determined using an enzyme-linked immunosorbent assay (ELISA). The study examined the relationships between cOC and cardiovascular/metabolic markers using inferential statistics and correlation coefficients. Multivariate linear analysis was performed to identify factors independently associated with the serum levels of cOC. Multivariate analysis revealed that MAP (B coefficient: 0.138, 95% CI: 0.028–0.247, *p* = 0.015) and the atherogenic index (B coefficient: 0.599, 95% CI: −0.039–1.161, *p* = 0.037) are independent predictors of cOC levels. A positive correlation was observed between cOC, PP, the atherogenic index, and HbA1, as well as an inverse correlation between cOC and HDL-c among the participants. Additionally, PP was positively correlated with HOMA-IR. Participants with elevated cOC levels showed higher MAP and atherogenic index values, indicating a potential connection between cOC and cardiovascular risk. cOC is independently associated with MAP and the atherogenic index, suggesting it may play a role in vascular remodeling and lipid metabolism. These results emphasize the importance of the bone–vascular axis in cardiovascular health and indicate that cOC might be a useful biomarker for assessing cardiovascular risk. Additional research is necessary to confirm these findings in larger, long-term studies and to investigate the mechanisms that connect cOC with cardiovascular outcomes.

## 1. Introduction

The interplay between bone metabolism and cardiovascular health has become an area of growing interest in recent years [1,2]. Bone-derived proteins, such as osteocalcin (OC), have been identified as potential biomarkers influencing both metabolic and cardiovascular pathways [2,3,4]. OC exists in two primary forms: carboxylated (cOC), which is retained in the bone matrix due to its high affinity for calcium hydroxyapatite, and undercarboxylated (ucOC), which circulates in the bloodstream and is associated with endocrine effects [1]. While ucOC has been extensively studied for its links to insulin sensitivity and metabolic disorders [1,2,3], the role of OC in cardiovascular physiology remains poorly understood. Total osteocalcin (tOC) has been identified in atherosclerotic plaques, with higher levels present in calcified plaques [4]. A positive correlation has been found between the number of endothelial precursor cells containing OC and the degree of coronary vessel calcification in patients with coronary heart disease [5]. Additionally, circulating monocytes expressing OC correlate with the amount of necrotic core and calcification degree in early stage atherosclerosis. Some researchers have also reported elevated tOC levels in patients with central arterial stiffness [6]. In their review, Tacey and colleagues concluded that the association between OC and vascular function in humans remains inconsistent. Some studies suggest that tOC is associated with either impaired or enhanced vascular function, while others report that neither tOC nor uOC affects vascular function [7]. OC may play an important role in atherosclerosis pathophysiology due to its high affinity for calcium [8] and its significant association with surrogate markers of arterial stiffness, such as pulse wave velocity [9] or serum lipids (e.g., HDL-c [10]). Emerging evidence suggests that cOC may influence vascular health through mechanisms linked to arterial stiffness [11,12]. This leads us to hypothesize a possible relationship between cOC and various cardiovascular and metabolic parameters. Indeed, plaque calcifications, which occur during the progression of atherosclerosis [13], have been linked to arterial stiffness [14] and may also be related to cOC. In this context, surrogate markers of vascular stiffness, such as mean arterial pressure (MAP), pulse pressure (PP), and the atherogenic index, have been shown to predict cardiovascular risk [15] and could potentially be associated with cOC. Understanding these associations is essential to unravel the complex interactions between bone-derived factors and cardiovascular disease (CVD). There are reports of a positive relationship between ucOC and blood pressure (both systolic and diastolic) [10]; however, no association between MAP, PP, the atherogenic index, and OC (in any form: cOC or ucOC) has been reported as a cardiovascular risk factor to date. This study investigates the relationship between cOC and surrogate markers of cardiovascular disease in a sample of the adult population from Western Mexico. By examining these relationships, this study contributes to the growing body of evidence on the bone–vascular axis and its implications for cardiovascular health.

## 2. Results

A total of eighty-six adults met the inclusion criteria. A total of 5 participants were excluded for the following reasons: newly diagnosed hypothyroidism (*n* = 2), calcium or calcitriol intake (*n* = 2), and lupus (*n* = 1), leaving 81 patients for analysis. The characteristics of the study population are detailed in Table 1.

There was a positive correlation between cOC, PP, the atherogenic index, and HbA1, as well as an inverse correlation with HDL-c among participants. Additionally, PP was positively correlated with HOMA-IR (Table 2).

**Figure 1:**
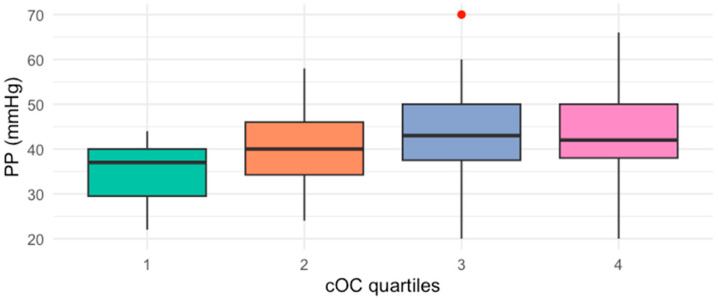
Median PP values by quartiles (Q1–Q4) of cOC (*p* = 0.013, Kruskal–Wallis test).

**Figure 2:**
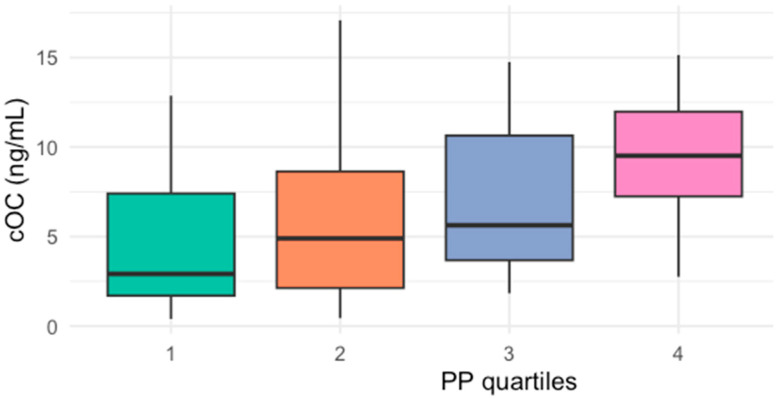
Median cOC serum levels by quartiles (Q1–Q4) of PP (*p* = 0.015, Kruskal–Wallis test).

When participants were classified by quartiles of cOC [cut-offs for 25th percentile: 2.64, 50th percentile: 5.32, and 75th percentile: 10.8 ng/mL], those with cOC serum levels in Q3 (43, IQR 36–50) and Q4 (42, IQR 37–50) had significantly higher PP than those in Q1 (37, IQR 28–40) (Figure 1). Similarly, when patients were classified by quartiles of PP, those in the highest quartile of PP had higher cOC serum levels (9.5, IQR 7–12 ng/mL) compared to those in the lowest quartile (4.3, IQR 4.1–5.7 ng/mL) (Figure 2).

A comparison of clinical and biochemical parameters between participants in Q3–Q4 versus those in Q1–Q2 of cOC revealed that individuals with higher serum levels of cOC had higher SBP and PP (Table 3); however, no other significant differences were observed.

A multiple linear regression analysis was performed to assess the association between cOC and multiple biomarkers. After adjusting for potential confounding variables, two significant biomarkers were associated with cOC, including MAP (*p* = 0.015) and the atherogenic index (*p* = 0.037) (Table 4).

## 3. Discussion

This study reveals important connections between cOC levels and crucial cardiovascular risk factors. A positive correlation between cOC, PP, the atherogenic index, and HbA1 levels was observed, while cOC was also inversely correlated with HDL-c levels, even after adjusting for creatinine. Furthermore, PP showed a positive correlation with HOMA-IR.

Patients in the highest concentration groups of cOC (Q3 and Q4) showed significantly elevated PP compared to those with lower serum cOC levels (Q1). Likewise, patients with the highest PP (Q4) had increased cOC levels when compared to those with the lowest PP (Q1).

When comparing subjects with cOC levels above the median (Q3 and Q4) to those with cOC levels below the median (Q1 and Q2), it was found that the former had higher values of SBP and PP.

In the multiple logistic regression analysis, both MAP and the atherogenic index were identified as independent predictors of cOC levels, indicating a possible role for cOC in vascular health and lipid metabolism. The independent link between cOC and MAP highlights how bone-derived proteins might affect blood pressure regulation. MAP represents the average pressure on arterial walls during a heartbeat and is a known predictor of cardiovascular risk. The positive correlation between cOC and the atherogenic index further supports the idea that cOC affects lipid metabolism. The atherogenic index, which is the logarithmic ratio of triglycerides to HDL cholesterol, serves as a strong indicator of cardiovascular risk and lipid imbalance. The inverse relationship between cOC and HDL-c found in this study reinforces this idea, suggesting that higher cOC levels may worsen dyslipidemia by lowering protective lipoproteins. These interactions between cOC and lipid metabolism could offer new perspectives on the relationship between bone health and cardiovascular disease.

There is limited research regarding the clinical implications of cOC. A model comprising age, HOMA-IR, and cOC explained 49% of the variability in PP. In contrast to this study, Polgreen and colleagues [16] reported higher SBP in patients with lower cOC; their patients were younger (18.6 years, range 17–22 years) than those in the present study and had lower mean SBP levels, as expected for their ages [16,17]. Thus, the differences in the findings of Polgreen’s study may be attributed to the differences in the populations studied, as this research showed that both age and cOC are positively correlated with PP.

The association between ucOC and other cardiovascular risk factors (such as SBP, DBP, HDL-c, LDL-c/HDL-c, and TC/HDL-c) has already been described by our group [10]. However, the relationship between cOC and surrogate markers of cardiovascular disease had not been previously evaluated. In a study conducted by Vasalle et al. [18], a significant relationship between increased levels of total OC (>75th percentile: 16.6 ng/mL) and higher Framingham risk scores was found, even after adjusting for confounding factors. This supports the hypothesis that OC is a bone-derived hormone independently associated with higher cardiovascular risk. The notion that bone-derived proteins are related to cardiovascular risk factors is not uncommon. In fact, several molecules produced by bone and released into circulation, such as osteopontin [19], osteoprotegerin [20], osteonectin [21,22], and matrix Gla-protein [23], have also been reported to be cardiovascular risk factors.

On the other hand, the relationship between PP and HOMA-IR found in the present study had been previously reported by Moon and Pergola, who observed a significant correlation between higher HOMA-IR and increased PP [24,25]. This might be explained by the physiological cardiovascular effects of insulin and the role of insulin resistance in hypertension and atherosclerosis [26].

Although we cannot establish a causal relationship between cOC and MAP, as well as between the atherogenic index and PP, possible explanations for this association include the following: firstly, cOC has a very high affinity for calcium hydroxyapatite in the bone, explained by the amino acid residues of γ-carboxyglutamic acid at positions 17, 21, and 24 of the protein [8]. Secondly, since intima and media calcification are known cardiovascular risk factors contributing to arterial stiffness [14], and PP serves as a surrogate marker for arterial stiffness, it is possible that bone-carboxylated proteins such as cOC accumulate in calcified sites of blood vessels when cOC levels rise to supraphysiological levels. Previous studies have demonstrated higher mRNA expression and protein accumulation of OC in calcified atheromas [26,27], as well as an increased release of OC from platelets in atherosclerotic lesions in patients with carotid artery occlusive disease [28]. Thirdly, preclinical studies have shown that OC induces adventitial remodeling by promoting the transformation of fibroblasts into myofibroblasts during vascular remodeling. Finally, a recent meta-analysis found a positive correlation between OC-positive cells, calcification, and atherosclerosis [29].

Several limitations of this study must be acknowledged. The most significant limitation is its cross-sectional design, which precludes the establishment of a causal relationship between cOC and MAP, as well as between the atherogenic index and PP. However, the Spearman correlation between PP and cOC should be taken with caution and should be retaken with a larger sample size, which would be desirable considering the same variables and thus verify this correlation. Additionally, a larger sample size would be desirable, along with adjusting for other potential confounders such as smoking status, menopausal status, and comorbid conditions such as diabetes mellitus and thyroid diseases.

## 4. Materials and Methods

### 4.1. Subjects and Ethical Considerations

This protocol was approved by the Ethics Committee of the University of Guadalajara (approval number: 18065), and it complied with the principles of the Helsinki Declaration. All participants provided written informed consent prior to their inclusion in the study.

All participants were adults (≥18 years of age) enrolled in the Detection and Treatment of Congenital and Acquired Metabolic Diseases program at the Health Science Center of the University of Guadalajara. A strict inclusion criterion required participants to belong to families who had resided in Western Mexico for at least three generations. Patients with any known rheumatic, thyroid, parathyroid, chronic kidney, or liver disease, as well as those with cancer or intestinal malabsorption syndrome, were excluded. Individuals taking medications known to affect OC synthesis or serum OC concentrations within the previous six months were also excluded. These medications included insulin, thiazolidinediones, glucocorticoids, contraceptives, vitamin D, calcium, calcitriol, bisphosphonates, thiazides, levetiracetam, and anticoagulants.

### 4.2. Clinical and Anthropometric Measurements

Demographic data, including age, sex, and comorbidities, were collected through interviews and physical examinations. All study participants underwent anthropometric measurements performed by trained examiners, which included weight (kg), height (cm), and body mass index (BMI; kg/m^2^). Body weight was measured to the nearest 100 g using a digital scale (Seca 354; SECA GmbH & Co. Kg, Hamburg, Germany), with participants wearing only light underwear and standing barefoot. Height was measured to the nearest 0.5 cm using a portable stadiometer (Seca 213; SECA GmbH & Co. Kg, Hamburg, Germany), ensuring the participant’s head was positioned in the Frankfurt plane. Both weight and height were measured twice, and the average of these measurements was used for subsequent calculations and analysis. BMI was calculated as weight in kilograms divided by the square of height in meters (kg/m^2^).

### 4.3. Biochemical Measurements

Blood samples were collected following a 12 h overnight fasting period and then centrifuged at 4500 rpm for 7 min to obtain serum. Glucose and lipid measurements were performed within two hours of sample collection. An aliquot of serum was stored at −70 °C for later quantification of cOC and insulin. Serum glucose and lipid levels were measured via spectrophotometry using the automated VITROS^®^ 5600 Immunodiagnostic system (Ortho-Clinical Diagnostics, Raritan, NJ, USA). Glycated hemoglobin was quantified via turbidimetry (Biosystems; Barcelona, Spain. Cat. No. 22047), while serum insulin levels were determined using a duplicate enzyme-linked immunosorbent assay (ELISA) (Genway Biotech, San Diego, CA, USA; Cat. No. GWB-D9BD0E). cOC was quantified using an immunosorbent enzyme assay (Takara Bio Inc, Otsu, Japan; Cat. No. MK111), with a coefficient of variation < 6%.

### 4.4. Statistical Analysis

Quantitative variables were assessed for normality using the Shapiro–Wilk test. Normally distributed variables were compared using a t-test and are expressed as the mean and standard deviation (SD). Non-normally distributed variables were compared using the Mann–Whitney U test and are expressed as the median and interquartile range (IQR). Quartile distribution was performed using the 25th, 50th, and 75th percentiles as cut-off values. Multiple groups were compared using the Kruskal–Wallis test. Qualitative variables were compared using the chi-square (χ^2^) test or Fisher’s exact test. Spearman’s coefficient was used to analyze the statistical correlation between cOC and quantitative parameters. A multiple linear regression analysis was performed to identify factors independently associated with the serum levels of cOC. A *p*-value of <0.05 was considered statistically significant. Data were analyzed using the Statistical Package for the Social Sciences (SPSS) version 21.0 (IBM Corporation; Armonk, NY, USA).

## Figures and Tables

**Table 1:** Sociodemographic, anthropometric, and biochemical characteristics of the participants (*n* = 81).

Characteristic	Median (IQR) *
Age (years)	47.0 (42–53)
Gender (male/female), %[n]	30.9 (25)/69.1 (56)
Diabetes, %[n]	33.3 (17)
Hypertension, %[n]	17.3 (14)
Weight (kg)	70.2 (63.3–79.3)
BMI (kg/m^2^)	26.7 (24.6–30.3)
Waist circumference (cm)	92.5 (85.18–101.13)
Body fat %	32.7 (24.48–40.50)
SBP (mmHg)	116 (110–124)
DBP (mmHg)	78 (72–82)
PP (mmHg)	40 (34–46)
cOC (ng/mL)	5.32 (2.64–10.80)
Total cholesterol (mg/dL)	182 (154.7–226)
HDL-c (mg/dL)	40.25 (34–48.71)
LDL-c (mg/dL)	105.5 (88.75–127)
Triglycerides (mg/dL)	120 (86.10–156.50)
Insulin (μU/mL)	9.96 (6.63–18.15)
HOMA-IR	3.12 (1.39–5.90)
HbA1c (%)	5.17 (4.71–7.17)
Creatinine (mg/dL)	1 (0.80–1.23)

BMI: body mass index; SBP: systolic blood pressure; DBP: diastolic blood pressure; PP: pulse pressure; cOC: carboxylated osteocalcin; HDL-c: high-density lipoprotein cholesterol; LDL-c: low-density lipoprotein cholesterol; HOMA-IR: homeostatic model assessment—insulin resistance; HbA1c: glycated hemoglobin. * Quantitative parameters are expressed as Median (25th–75th IQR), whereas qualitative parameters are expressed as %[n].

**Table 2:** Spearman (ρ) correlation coefficients of cOC and PP with anthropometric and metabolic parameters.

Parameter	cOC	PP
cOC (ng/mL)	-----	0.342 **
PP (mmHg)	0.342 **	-----
Total cholesterol (mg/dL)	−0.122	0.019
HDL-c (mg/dL)	−0.239 *	0.005
LDL-c (mg/dL)	0.097	−0.044
Triglycerides (mg/dL)	0.083	0.079
Insulin (μU/mL)	0.007	0.208
HOMA-IR	0.079	0.285 *
HbA1c (%)	0.346 *	0.230
Atherogenic index	0.224 *	−0.027

BMI: body mass index; SBP: systolic blood pressure; DBP: diastolic blood pressure; PP: pulse pressure; cOC: carboxylated osteocalcin; HDL-c: high-density lipoprotein cholesterol; LDL-c: low-density lipoprotein cholesterol; HOMA-IR: homeostatic model assessment—insulin resistance; HbA1c: glycated hemoglobin. * *p* < 0.05; ** *p* < 0.01.

**Table 3:** Comparison of clinical and biochemical characteristics of participants by cOC quartiles.

Characteristic	cOC <5.32 ng/mL (Q1–Q2)	cOC >5.32 ng/mL (Q3–Q4)	*p*-Value *
Age (years)	45 (40.75–49.25)	49 (45–54.5)	0.139
Gender (%male/female)	34/66	25/75	
Weight (kg)	73 (62.48–86.15)	68.9 (63.65–75.4)	0.366
BMI (kg/m^2^)	27 (25.2–30.6)	26.05 (23.73–29.7)	0.314
Waist circumference (cm)	96 (85.5–105.75)	88.88 (84.53–95.38)	0.402
Body fat %	33.95 (27.33–40.80)	31.35 (22.78–38.05)	0.435
SBP (mmHg)	110 (108–120)	120 (110–135)	<0.001
DBP (mmHg)	78 (70–80.5)	80 (72–87)	0.557
PP (mmHg)	39 (30–42)	42 (37–87)	<0.001
Total cholesterol (mg/dL)	177.5 (161.25–216.63)	190.0 (149.05–238.35)	0.711
HDL-c (mg/dL)	41 (36.25–52.8)	38.6 (31.58–46.52)	0.311
LDL-c (mg/dL)	102.5 (87.5–126.5)	108.5 (89.5–128.25)	0.965
Triglycerides (mg/dL)	108.5 (84–148.5)	126.6 (88.13–182.95)	0.601
Insulin (μU/mL)	10.12 (6.03–23.3)	9.96 (7.27–14.49)	0.333
HOMA-IR	2.84 (1.28–5.61)	3.35 (1.92–6.07)	0.323
HbA1c (%)	4.89 (4.61–6.87)	5.79 (4.98–7.17)	0.976

BMI: body mass index; SBP: systolic blood pressure; DBP: diastolic blood pressure; PP: pulse pressure; cOC: carboxylated osteocalcin; HDL-c: high-density lipoprotein cholesterol; LDL-c: low-density lipoprotein cholesterol; HOMA-IR: homeostatic model assessment—insulin resistance; HbA1c: glycated hemoglobin. * Mann–Whitney U test.

**Table 4:** Multivariate linear analysis for factors independently related to cOC.

Characteristic	B Coefficient	IC95%	*p*-Value
Age (years)	0.033	−0.140 to 0.207	0.700
MAP (mmHg)	0.138	0.028 to 0.247	0.015
HbA1c (%)	−0.099	−1.128 to 0.930	0.847
HOMA-IR	0.142	−0.313 to 0.598	0.532
BMI (kg/m^2^)	−0.224	−0.501 to 0.052	0.109
Atherogenic index	0.599	−0.039 to 1.161	0.037

## Data Availability

The Excel file database used to support the findings of this study is available from the corresponding author upon request.
